# Nature‐Positive Materials Engineering: Carbon Electrodes from Satoyama Biomass

**DOI:** 10.1002/tcr.202600007

**Published:** 2026-05-02

**Authors:** Yuta Nakayasu

**Affiliations:** ^1^ Frontier Research Institute for Interdisciplinary Sciences Tohoku University Sendai Japan

**Keywords:** biochar, biomass, carbon electrodes, nature‐positive, satoyama

## Abstract

Forest overuse is widely recognized globally, yet in Japan, the underuse of secondary forests is increasingly degrading satoyama ecosystems. At the same time, electrochemical energy technologies remain strongly dependent on mined graphite and trace or precious metals. In this Personal Account, I examine whether sustainably managed satoyama biomass can serve as a locally available carbon feedstock for electrochemical energy storage and conversion. Rather than surveying biomass utilization in general, I focus on the structure–property–performance relationships of wood‐derived and satoyama‐relevant carbons in batteries, supercapacitors, electrocatalysis, and bioelectrochemical systems. The available evidence indicates that this proposition is feasible, provided that its scope is clearly defined: underused satoyama biomass can already function effectively as activated carbons, hard carbons, graphitized carbons, and catalyst supports, although important bottlenecks remain in feedstock variability, volumetric performance, process standardization, and scale‐up. By linking coppicing and thinning cycles with carbonization and device fabrication, this framework may reduce mining dependence, support biodiversity‐oriented forest stewardship, and retain value within regional material cycles.

## Graphical abstract

Nature‐positive materials engineering can link satoyama forest stewardship with the design of carbon electrodes for batteries, supercapacitors, and electrocatalysis. By turning underused firewood and charcoal resources into functional carbons, this approach reduces mining footprints while supporting biodiversity, landscape resilience, and regional value chains.

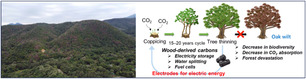

## 1 Introduction: From Forest Underuse to Nature‐Positive Energy Materials

### 1.1 Global Changes and Utilization of Forests

Forests provide numerous ecosystem services, broadly classified into four categories: provisioning, regulating, cultural, and supporitng [1]. Supporting services are basic ecosystem functions, while provisioning, regulating, and cultural services directly benefit humans. Among these services, the provisioning of woody materials remains economically important, including fuelwood, construction timber, and industrial feedstocks [2]. Table 1 summarizes changes in forest growing stock and carbon storage across continents from 1990 to 2020 based on the FAO FRA 2020 reporting framework. Here, carbon stock in biomass denotes carbon stored in above‐ and below‐ground living biomass, whereas “total forest carbon stock” includes biomass carbon plus dead wood, litter, and soil organic matter. All values were taken directly from the FAO reporting tables and were not recalculated by the author [3]. Across the FRA 1990–2020 window, temperate regions such as Europe and North/Central America generally increased in growing stock and biomass carbon, whereas South America and Africa showed overall declines. Thus, regional divergence—rather than a uniform global trend—is the key message relevant to this Account.

**TABLE 1:** Changes in forest stock from 1990‒2020 on each continent [3].

Region		1990	2000	2010	2020
Total Africa	Growing stock (billion m^3^)	87.64	84.36	80.99	76.41
	Carbon stock in biomass (Gt)	58.74	56.29	53.74	50.57
	Total carbon stock (Gt)	94.27	90.14	85.91	80.89
Total Asia	Growing stock (billion m^3^)	51.59	54.29	58.2	62.5
	Carbon stock in biomass (Gt)	34.08	35.09	36.16	37.55
	Total carbon stock (Gt)	77.09	78.56	81.94	84.78
Total Europe	Growing stock (billion m^3^)	104.28	108.09	113.06	116.23
	Carbon stock in biomass (Gt)	45.14	47.57	51.23	54.57
	Total carbon stock (Gt)	158.75	162.47	168.08	172.45
Total North and Central America	Growing stock (billion m^3^)	90.35	91.81	93.25	95.07
	Carbon stock in biomass (Gt)	39.41	40.37	40.9	41.63
	Total carbon stock (Gt)	143.18	144.13	145.57	146.12
Total Oceania	Growing stock (billion m^3^)	18.71	18.71	18.8	18.87
	Carbon stock in biomass (Gt)	13.94	13.87	13.86	13.88
	Total carbon stock (Gt)	33.34	33.11	33.08	33.06
Total South America	Growing stock (billion m^3^)	207.19	199.02	190.75	187.45
	Carbon stock in biomass (Gt)	106.5	102.38	98.21	96.33
	Total carbon stock (Gt)	161.76	154.92	147.92	144.85
World	Growing stock (billion m^3^)	559.76	556.28	555.05	556.53
	Carbon stock in biomass (Gt)	297.81	295.57	294.1	294.53
	Total carbon stock (Gt)	668.39	663.33	662.5	662.15

Historically, forests have been depleted for two main reasons: (1) harvesting for daily needs (fuel, construction materials, tools) and (2) clearing land for agriculture and urbanization. In Europe, forest cover declined as population density grew and societies developed [4]. After experiencing deforestation and related crises (wood shortages, soil erosion, degradation), many developed countries enacted forest management policies to shift from net forest loss to net expansion. In developing regions such as Africa and South America, however, rapid development through use of local timber, forest conversion, and cash‐crop cultivation continues to reduce forest area. Thus, restoring overexploited forests globally remains imperative.

### 1.2 Changes and Utilization of Secondary Forests in Japan

Turning to local issues, Japan faces an emerging problem of forest ‘underuse.’ Although deforestation occurred in the past, Japan established regulations and management systems as early as the 17th century to address wood shortages; active afforestation programs and silvicultural techniques followed in the 18th century [5]. After World War II, extensive reforestation was conducted (e.g., large‐scale planting of Cryptomeria japonica and Chamaecyparis obtusa). However, since the 1970s, the demand for domestic timber has plummeted, leaving many cedar plantations and coppice forests formerly used for firewood, and charcoal underutilized [6]. As a result of this underuse, problems have arisen—for example, neglected forests have deteriorated, and crop damage by wild herbivores has increased—exacerbated by rural depopulation and a lack of active forest management [7].

The modern concept of ‘sustainability’ in forestry can be traced back to 17th‐century experts who introduced the idea of sustainable yield to counter forest decline [8]. In recent years, the traditional term satoyama has regained attention in Japan to refer to forests that require active management. Because the term lacks a precise definition, it can cause confusion in scientific discussions. In this Personal Account, I define satoyama as secondary forests and grasslands near human settlements that require continuous human stewardship to sustain their ecological functions.

I also use ‘nature‐positive’ to mean more than just ‘low‐carbon’ – it describes materials and systems that actively reduce pressures on ecosystems (for example, shrinking mining footprints), support biodiversity and landscape functions through stewardship, and embed value within local, regenerative resource cycles.

Historically, satoyama landscapes expanded during the medieval period, and by the early modern era, resource use in these areas had reached extreme levels [9]. In mountainous regions, harvesting firewood and charcoal from nearby satoyama forests has been practiced since ancient times. During the early modern period, commercial demand for firewood and charcoal grew even in major cities, prompting various regulations to ensure sustainable resource use in satoyama [10].

### 1.3 Coppicing and Oak Wilt in Secondary Forests in Japan

**FIGURE 1:**
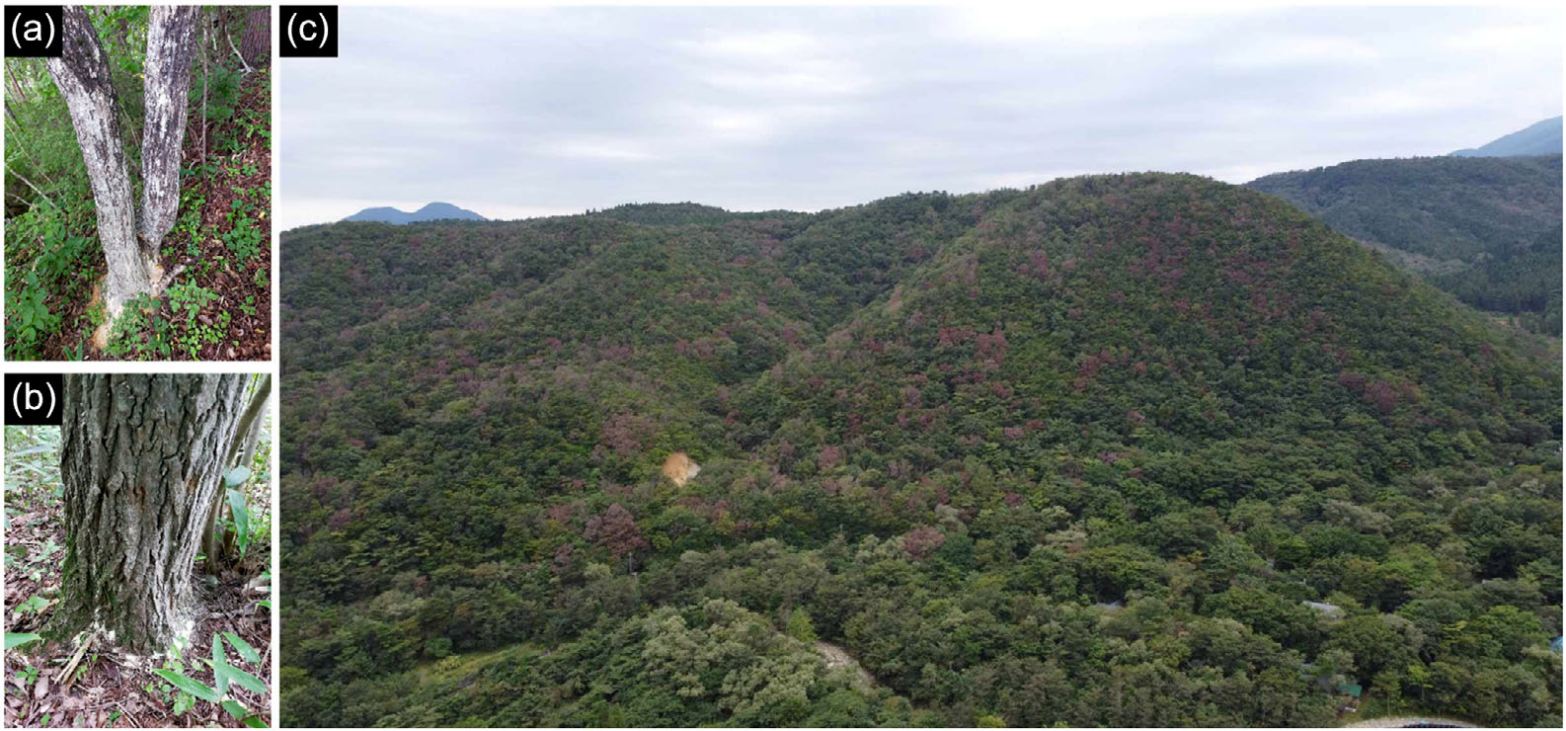
(a, b) Firewood and charcoal forest in Miyagi prefecture immediately following an insect infestation of Quercus serrata. (c) Japanese oak wilt in a satoyama forest in Miyagi prefecture. Photographs in panels (a) and (b) were taken by the author; panel (c) was kindly provided by Mr. Ogasawara.

Coppicing is a traditional practice for managing deciduous trees whereby, after harvest, new shoots regenerate from the stump without replanting. For example, a sustainable wood supply can be maintained by local residents harvesting oak trees every 15–20 years in rotation [11]. However, while local communities once depended on satoyama forests for fuel, demand for firewood and charcoal has sharply declined in recent decades. Consequently, satoyama oak forests (dominated by Fagaceae species such as Quercus, Castanea, Castanopsis, and Lithocarpus) have grown older and overstocked—notably, the principal oaks Quercus crispula and Q. serrata now often reach diameters of 40–50 cm at breast height (DBH) [12]. These larger, aging oaks are highly susceptible to infestation by ambrosia beetles, which carry the pathogenic fungus Raffaelea quercivora that causes Japanese oak wilt (JOW) [13, 14]. Oak stands afflicted by JOW lose many ecosystem services: they sequester less carbon, suffer more erosion, and harbor lower biodiversity than healthy stands [15]. Figure 1a,b show a firewood/charcoal forest immediately after Q. serrata trees were infested, and Figure 1c shows a satoyama oak with JOW. An infestation is readily identified by piles of frass (wood powder) at the base of the trunk. (Notably, in Figure 1c—taken in September before normal autumn leaf color change—the dead leaves turned red, making the infection easy to spot.)

Active management through thinning and coppicing can increase understory biodiversity [16], enhance carbon sequestration, and potentially reduce JOW risk [17]. Nevertheless, it remains difficult to motivate people today to carry out long‐term satoyama stewardship. Therefore, in order to sustain these forests, the use of firewood and charcoal resources should be encouraged, along with support for the cultural and economic roles that firewood plays as a local fuel.

### 1.4 Wood Utilization for Energy

In 2022, Japanese household energy consumption from fossil fuels (e.g., LP gas, city gas, kerosene) remained substantial and was roughly comparable to that from electricity [18]. In addition, only a small fraction of households used wood‐burning heating devices in Japan [19]. Recent energy‐price volatility has renewed attention to alternative heating options, although household use of wood‐burning devices remains limited in Japan [19, 20]. Historically, before widespread electrification, a large share of household energy demand was associated with heat‐related uses such as cooking, water heating, and space heating; today, even heating is increasingly supplied by electricity (e.g., all‐electric homes). Although woody biomass power generation is often labeled ‘carbon‐neutral,’ life‐cycle assessments indicate it is not necessarily so [21]. Therefore, beyond simply burning wood as fuel, it is worth exploring ways to use wood that retain the carbon in long‐lived materials and thereby reduce broader ecological pressures—an approach more naturally aligned with a nature‐positive framework.

To position the electrochemical route within the broader context of wood utilization, Table 2 summarizes representative woody‐biomass utilization pathways relevant to energy use and carbon retention. The electrochemical route differs from direct fuel use in that carbon can be retained in functional materials for longer periods while potentially displacing mined inputs. For this reason, the following sections focus on carbonization and materials design rather than combustion alone.

**TABLE 2:** Representative woody‐biomass utilization pathways relevant to energy use and carbon retention.

Pathway	Typical use	Carbon fate	Main implication	Representative refs.
Direct fuelwood/charcoal use	Household heat and cooking	Rapid carbon release during combustion	Historically central to satoyama resource use but associated with low value addition and limited modern uptake	[2, 10, 19]
Woody biomass power generation	Electricity and/or heat generation	Rapid carbon release during energy generation	Can process relatively large biomass volumes, but its “carbon neutrality” is strongly dependent on life‐cycle boundary conditions	[21]
Biochar/soil amendment	Soil amendment and carbon management	Partial longer‐term carbon retention in soil	A relevant carbon‐retaining alternative, although cost‐effectiveness is strongly context‐ and region‐dependent	[22]
Wood‐derived electrochemical carbons	Batteries, supercapacitors, electrocatalysis, and bioelectrochemical systems	Carbon retained in longer‐lived functional materials during device use	Higher value‐added utilization route and the central focus of this Account, although process control, standardization, and scale‐up remain necessary	[23]

## 2 Electrode Materials for Renewable Energy Devices

### 2.1 Mined Resources in Electrodes and Decreased Biodiversity

Electrodes in many renewable energy devices (batteries, fuel cells, electrolyzers) often depend on mined resources, including scarce precious metals such as platinum and ruthenium. These resources are very expensive due to their low abundance and difficult supply. For example, lithium‐ion battery anodes use either artificial graphite (made from petroleum pitch via an energy‐intensive process) or natural graphite (mined in limited regions and refined with chemical‐ and energy‐intensive methods) [24, 25, 26]. Because the relevant descriptors differ across device classes, Table 3 compares device‐appropriate benchmarks—capacity and initial Coulombic efficiency for battery anodes, capacitance or energy density for supercapacitors, and overpotential/half‐wave potential plus durability for catalytic systems. This comparison also clarifies that satoyama biomass is most immediately promising not as a universal substitute but as a feedstock for selected carbon functions such as activated carbons, hard carbons, graphitized carbons, catalyst supports, and bioelectrochemical electrodes [37, 38, 39, 40].

**TABLE 3:** Quantitative benchmark of representative woody biomass‐derived carbons for electrochemical energy storage and conversion.

Feedstock	Carbon/processing strategy	Device and electrode role	Key quantitative performance	Main implication for satoyama design	Ref.

Hardwood sawdust	Fe‐catalyzed laser graphitization to biochar graphite	Li‐ion battery anode	~ 357 mAh g^−1^ at C/2; ~94% Coulombic efficiency; ~1% capacity loss after 100 cycles	Wood‐derived graphite can approach battery‐grade graphite gravimetrically, but macroporosity still limits volumetric capacity	[27, 28]
Fungus‐pretreated basswood	Hierarchical hard carbon obtained after fungal pretreatment and carbonization	Na‐ion battery anode	242.3 mAh g^−1^ at 0.2 A g^−1^; ICE 88.2%; 93.9% retention after 200 cycles	Biological pretreatment can tune tortuosity and porosity before carbonization	[29]
Oak‐derived hard carbon + quinones	CO_2_‐activated oak hard carbon for quinone confinement	Aqueous organic redox capacitor/battery‐type carbon electrode	AQ utilization 97.6% (250.9 mAh g^−1^); 91.0% retention after 1,000 cycles for DCAQ anode	Satoyama‐relevant oak is already linked to functional organic‐carbon hybrid electrodes	[30]
Chinese fir wood scraps	Wood‐derived thick carbon electrode with activated porous structure	Aqueous supercapacitor electrode	285.6 F g^−1^; 38.0 mWh cm^−3^	Shape‐preserving wood carbons can work as monolithic thick electrodes for safe aqueous systems	[31]
Wood carbon after fungal pretreatment + MnO_2_	Biological pore regulation followed by MnO_2_ loading	Aqueous supercapacitor electrode	3,395 mF cm^−2^ at 1.0 mA cm^−2^; 88.6% retention after 2,000 cycles	Hybridization of tuned wood pores with pseudocapacitive phases is effective for areal devices	[32]
Graphitized wood‐derived carbon	Pt–Ni recombination/nickel phosphate on graphitized wood carbon	HER electrocatalyst support	10 mV overpotential at 10 mA cm^−2^; 30 mV dec^−1^; >120 hr durability	Wood‐derived graphitic supports can reduce reliance on expensive carbon nanostructures	[33]
Paulownia wood	PB‐assisted Fe_3_C nanoparticles + N‐doped wood‐derived carbon (Fe_3_C@NPW)	ORR catalyst/ Zn–air battery cathode	*E*_1/2_ = 0.87 V vs. RHE; 804.4 mAh g_Zn_^−1^; 78 mW cm^−2^; 780 cycles	Wood‐derived carbons can host dual active sites and exceed Pt/C in alkaline ORR	[34]
Oak white charcoal	Minimally processed charcoal biocathode	Methanogen biocathode/ microbial fuel cell	≈4.0‐fold more electrons and ≈1.7‐fold more CH_4_ than carbon felt	Minimally processed carbons are also valuable in bioelectrochemical systems	[35, 36]

Because of high prices, supply chain risks [41], poor labor conditions in mining, and geographical concentration of resources [42], it is highly desirable to minimize the use of such mined resources. One report notes that current mining activities pose a biodiversity risk comparable to climate change [43]. Given that ~ 1,179 species (about 5% of evaluated species) are threatened by both climate change and mining, measures are needed to mitigate the impact of mining on ecosystems. If future mining operations cause similar biodiversity losses, this threat could intensify—especially since demand for certain energy materials (e.g., cobalt, lithium) is projected to increase by 500–900% by 2050 [44]. Moreover, as ore quality declines, mining will require more drilling and generate more waste, further worsening biodiversity loss [45]. In 2021, the G7 nations recognized this issue in their ‘G7 2030 Nature Compact,’ committing to conserve at least 30% of land and sea by 2030 to halt and reverse biodiversity loss (the ‘30 × 30’ goal). This commitment underscores that, in line with a nature‐positive approach, we should prioritize electrode materials made from abundant, widely available elements as alternatives.

### 2.2 Carbon‐Based and Organic Materials for Electrodes Instead of Critical or Precious Metals

There are promising nonmetal alternatives for electrodes. For example, certain organic compounds have been explored as LIB cathode materials [46, 47]. Likewise, considerable effort has been devoted to producing graphite from biomass for use as LIB anodes [48, 49]. In electrocatalysis, metal‐based catalysts for the hydrogen and oxygen evolution reactions (HER and OER) can be supported on carbon materials, thereby reducing the required precious‐metal loading [50, 51]. Similarly, in hydrogen fuel cells, carbon supports can help cut down the amount of platinum needed for the hydrogen oxidation reaction [52]. Notably, emerging metal‐free carbon catalysts have in some cases outperformed platinum in the oxygen reduction reaction (ORR) [53, 54], and complexes such as iron phthalocyanine or iron azaphthalocyanine have also been effective ORR catalysts [55, 56, 57, 58].

### 2.3 Why Satoyama Biomass Merits Consideration as an Electrode Feedstock

Most carbon materials used in energy devices today still come from fossil resources or energy‐intensive mining. This work began with a practical question: Can underutilized satoyama biomass be upgraded from an underused forest carbon resource into a renewable feedstock for selected electrode functions? This question spurred my group to investigate whether we could produce carbon materials directly from satoyama wood and charcoal. Our initial experiments were not immediately successful. We discovered that porous carbons made from natural biomass showed large variability in pore size and surface chemistry, making them ill‐suited for electrodes where precise control of microporosity, heteroatom content, and electronic conductivity is crucial. These setbacks forced us to rethink our approach. We identified two complementary strategies moving forward: (1) use mimally processed biochar materials in bioelectrochemical systems (such as microbial fuel cells or wastewater‐treatment electrodes), where a hierarchical pore structure is beneficial and fine‐tuning is not critical; and (2) develop new synthesis protocols to achieve tightly controlled structures (through pore engineering and catalytic graphitization) for highly ordered carbons in batteries and electrocatalysts. This shift in approach ultimately shaped the research directions described in Sections 3 and 4.

**FIGURE 2:**
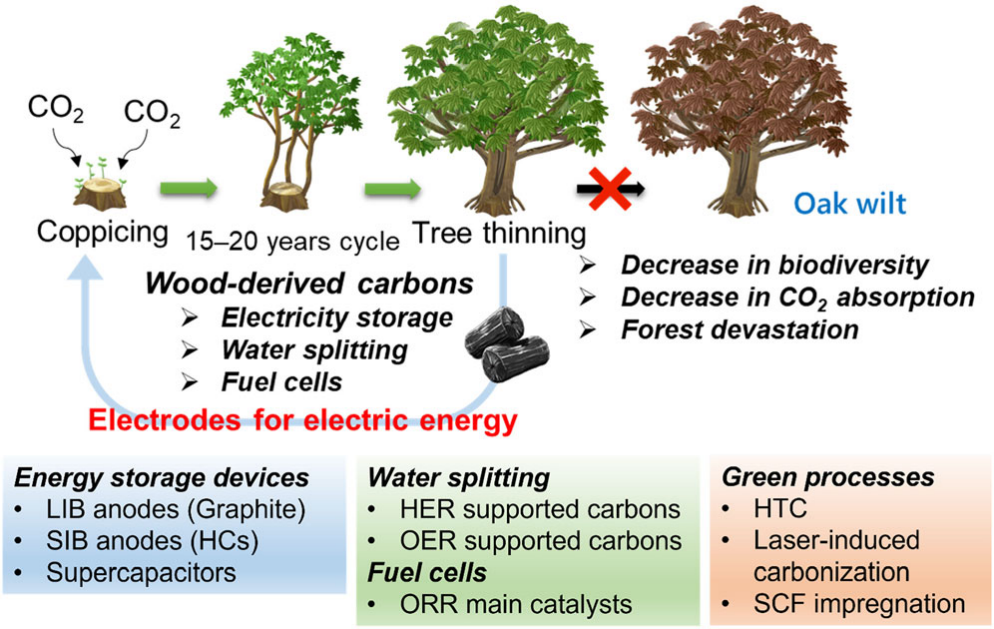
Uses of carbon derived from felled wood as part of the sprouting and regeneration cycles, and potential electrode materials for each energy device and their green fabrication processes.

We envisioned a cyclical system in which carbon‐rich tree species—managed via periodic coppicing and thinning—are sustainably harvested and converted into electrode materials, all while supporting biodiversity and local landscape maintenance (Figure 2). In recent work, we have begun prototyping this concept by explicitly linking forest management cycles to materials fabrication. This strategy addresses two intertwined challenges: the degradation of neglected secondary forests and the global reliance on mined resources for energy storage and generation. By aligning material synthesis with ecological stewardship, we found a pathway that is both technologically promising and regionally meaningful. This approach now forms the conceptual backbone of our subsequent developments in LIB/SIB anodes, organic batteries, and carbon‐supported electrocatalysts (detailed in later sections).

The cell walls of wood have a consistent makeup across species (~40–45% cellulose, 15–25% hemicellulose, 25–30% lignin) [59]. Biochar from wood generally contains less ash and fewer impurities than biochar from other biomass sources, which is advantageous for materials synthesis [60]. It also has a higher fixed‐carbon fraction compared to other biomass feedstocks [60].

While biomass is often considered mainly as a fuel, I chose to focus on carbonization and materials design—a fundamentally different mode of resource use. The rationale is both materials‐based and systems‐based. Structurally, wood's natural hierarchical architecture can be preserved and modified through controlled carbonization (whereas simple combustion would destroy it). Socially and from a regional‐systems standpoint, converting local biomass into functional materials extends the resource's lifecycle and retains value within the local economy. For these reasons, wood‐derived carbons are attractive not only as renewable feedstocks but also as platforms for locally embedded materials design.

In terms of applications, using biochar as a soil amendment is often not cost‐effective in high‐income countries (e.g., the US or UK), but it can be economical in low‐income rural communities where it replaces expensive fertilizers, boosts crop yields, and reduces labor costs [22]. On the other hand, using woody biomass‐derived carbon in electrochemical devices may offer a higher value‐added use in some contexts; however, this point requires dedicated techno‐economic and life‐cycle analysis. Wood‐derived carbons offer a naturally hierarchical pore structure, generally low ash content, and tunable surface chemistry; these features make them attractive electrode platforms, especially where pore architecture and shape‐preserving carbonization are advantageous [23]. As summarized in Table 4, satoyama should not be treated as just another biomass source. Its distinguishing feature is that feedstock generation is inseparable from active secondary‐forest stewardship, which introduces both advantages (local circularity, biodiversity support, regional value retention) and technical challenges (species heterogeneity, seasonality, and standardization). Taken together, the literature and our own studies suggest a qualified yes: underutilized satoyama trees can serve as renewable carbon sources for selected electrode roles—especially activated carbons, hard carbons, graphitized carbons, and catalyst supports. However, this is not yet a universal substitute for conventional electrodes, because feedstock variability, volumetric performance, process standardization, and scale‐up remain unresolved.

**TABLE 4:** Why satoyama‐derived biomass is distinct from generic wood feedstocks used in previous electrode studies.

Aspect	Typical wood feedstocks in previous literature	Satoyama‐derived biomass/carbon	Implication for electrode design in this Account

Resource origin	Industrial wood scraps, sawdust, or plantation timber	Coppiced/thinned secondary‐forest biomass, firewood or charcoal residues, oak‐wilt removals	Links materials production to forest stewardship rather than only waste valorization
Species composition	Often single species and relatively uniform feedstocks	Mixed broadleaf species with site‐ and management‐dependent variability	Requires species screening, grading, and clearer materials specifications
Management logic	Byproduct utilization after timber processing	Biomass generated through thinning, coppicing, and restoration‐oriented interventions	Electrode production can be coupled to biodiversity maintenance and landscape care
Structural inheritance	Hierarchical wood channels preserved during carbonization	Same advantage, but with broader variability among stem/branch/charcoal‐derived precursors	Device class should be matched to the degree of feedstock controllability
Supply‐chain scale	Relatively centralized and high‐volume	Distributed, seasonal, and regionally limited	Modular regional processing may be more realistic than mega‐scale centralized production
Most suitable near‐term roles	Broad range, depending on process control	Activated carbons, hard carbons, catalyst supports, and bioelectrochemical electrodes; graphitic anodes only after stricter control	Supports a cautious conclusion: promising for selected roles, not yet universal
Main unresolved bottleneck	Scale‐up and volumetric performance	Scale‐up, volumetric performance, plus feedstock heterogeneity and logistics	Standardization, LCA/TEA, and local supply‐chain design must be discussed explicitly

Published examples that are explicitly satoyama‐relevant remain limited but instructive. In our own work, oak from firewood/charcoal systems has already been used for quinone‐based aqueous organic electrodes and minimally processed charcoal biocathodes, while related studies on wood‐derived graphitic or hard carbons clarify which additional levels of control are required for higher‐performance battery and catalytic applications. This imbalance in the literature is itself informative: the near‐term opportunity for satoyama biomass lies first in activated carbons, hybrid organic–carbon electrodes, catalyst supports, and bioelectrochemical electrodes, rather than in universal replacement of all commercial carbons.

## 3 Energy‐Related Devices Using Local Woody Biomass‐Derived Carbons

Unlike several recent reviews that comprehensively survey biomass‐derived electrode materials across diverse systems [61, 62, 63], this section does not aim to provide an exhaustive overview. Instead, it highlights representative wood‐derived and satoyama‐relevant examples across batteries, supercapacitors, electrocatalysis, and bioelectrochemical systems to clarify where woody biomass already performs well and where major bottlenecks remain. In the last part, contributions from our research group are summarized in this context, demonstrating how process design, pore engineering, and device integration can be leveraged to overcome these bottlenecks.

### 3.1 Secondary Batteries

Two practical routes can currently be identified for integrating wood‐derived materials into rechargeable batteries. First, replace conventional anodes (e.g., graphite in Li‐ion batteries and hard carbon in Na‐ion batteries, the dominant battery types today) with wood‐derived carbon anodes. Second, replace both the anode and cathode with wood‐derived carbon‐based components.

**FIGURE 3:**
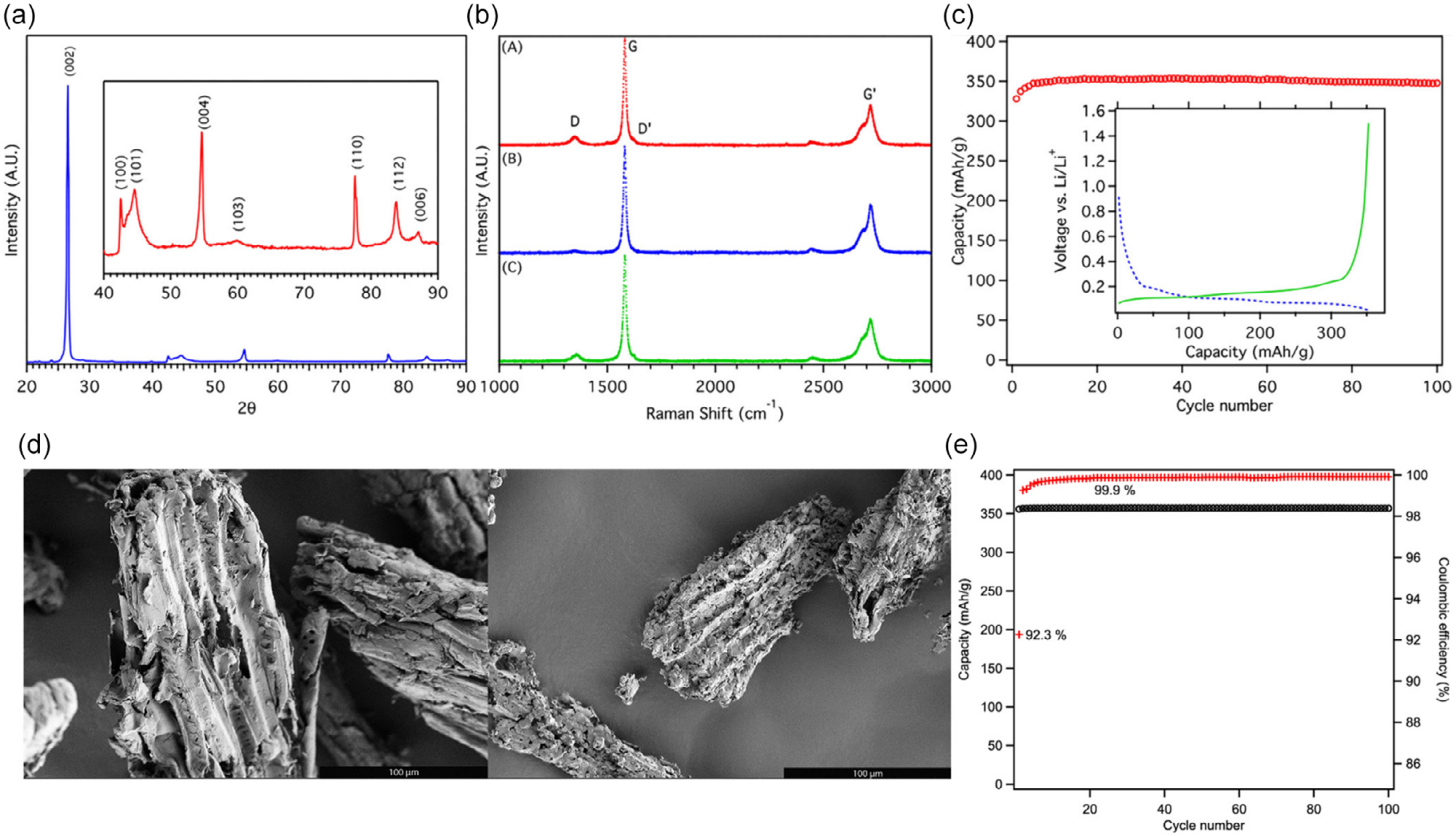
(a) XRD pattern (blue) of ∼5 µm biochar graphite (BCG). Inset is scaled to show lower intensity reflections (red). (b) Raman spectra of (A, red) SFG6, (B, blue) 0.5–1 mm BCG, and (C, green) ∼5 μm BCG. (c) Gravimetric capacity of a ∼5 μm BCG (made from −325 mesh Fe) anode demonstrating excellent Li‐ion capacity (357 mAh·g^−1^, red circles), comparable to that of commercial Li‐ion battery grade graphite with only a 1% capacity loss over 100 charge/discharge cycles at a C/2 rate. Inset is its charge (blue dashed line)/discharge (green line) profile, which is, again, nearly identical to that of commercial graphite. The images in (a–c) were reproduced from a previous study [27] with permission from ACS Publications, Copyright 2018. (d) SEM images of sawdust char (left) and BCG made from sawdust char (right) showing preservation of wood‐like morphology during graphitization. (e) Capacity (black circles) and Coulombic efficiency (red crosses) of BCG made from 230 to 140 mesh sawdust. The images in (d) and (e) were adapted from Ref. [28], licensed under the Creative Commons Attribution 4.0 International License (CC BY 4.0).

**FIGURE 4:**
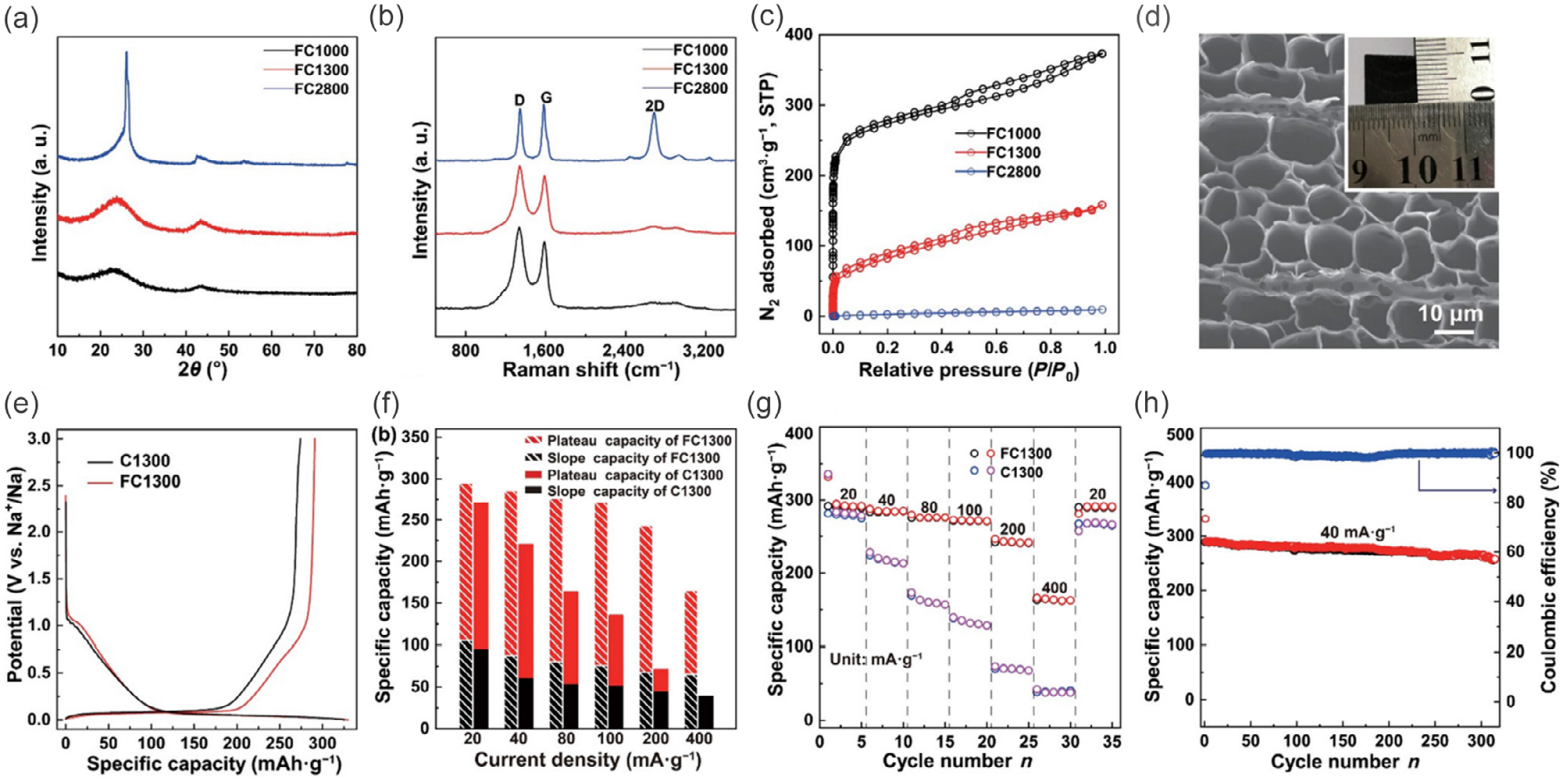
Structural properties of carbon materials: (a) XRD patterns; (b) Raman spectra; (c) N_2_ adsorption‒desorption isothermal curves of FC1000/1300/2800; and (d) SEM image of FC1300 (inset is a photograph of FC1300). Electrochemical performances: (e) initial galvanostatic charge–discharge curves; (f) distribution of sloping discharge capacity and plateau discharge capacity at different current densities; and (g) rate performance of FC1300/C1000. (h) Cycling performance of FC1300 at 40 mA·g^−1^. The images were adapted from a previous study [29] with permission from Springer, Copyright 2023. The wood‐derived carbon material that was treated with fungi is abbreviated as FC, and the untreated material is abbreviated as C.

Graphite has a theoretical capacity of 372 mAh·g^−1^ (LiC_6_). Recent work on synthesizing highly crystalline carbon from biomass has focused on iron‐based catalysts [64, 65, 66, 67]. Banek et al. [27] succeeded in producing ‘biochar graphite’ from hardwood sawdust via iron‐catalyzed CO_2_ laser graphitization (see Figure 3a–c). In a follow‐up study, they replaced the CO_2_ laser with a diode laser and optimized the process, achieving ~94% Coulombic efficiency and ~ 357 mAh·g^−1^ capacity at C/2 from wood‐derived graphite (Figure 3d,e) [28]. While the capacity and efficiency of such graphitic carbons can match commercial graphite, their extensive macroporosity limits volumetric capacity [66]. SIB (sodium‐ion battery) development has also advanced considerably. Various wood‐based hard carbons (HCs) have been successfully used as SIB anodes [68, 69, 70]. Although the Na^+^ storage mechanism in HCs is still debated [71], it is significant that natural wood's hierarchical structure is retained in the carbonized hard carbon. This multi‐scale porosity shortens ion diffusion paths and increases the number of active storage sites, thereby enhancing capacity [72]. Furthermore, the performance of wood‐derived HCs can be improved via heteroatom doping, pore size control, or filler infusion [73]. For example, Wang et al. [29] pretreated basswood with fungi, then carbonized it at 1,000°C, 1,300°C, and 2,800°C to create HCs with hierarchical porosity (Figure 4a–d). The sample treated at 1,300°C (denoted FC1300, fungus‐conditioned carbon) delivered a high rate capacity of 242.3 mAh·g^−1^ at 0.2 A·g^−1^, retained 93.9% of its capacity after 200 cycles at 40 mA·g^−1^, and showed an improved initial Coulombic efficiency of 88.2% (Figure 4e–h) [29], outperforming the untreated wood carbon (C1300) in all metrics.

Despite these advances, further research must balance structural stability with energy performance – for instance, by tuning surface area and developing low‐cost extraction methods for wood‐based materials. Notably, the energy storage cost efficiency of wood carbons is already promising: a half‐cell SIB made from glucose‐derived hard carbon can deliver ~ 199 mAh per US dollar, ~1.78× higher capacity‐per‐cost than a typical LIB [74].

Within this context, our group has explored the synthesis and application of biomass‐derived graphitic and hard carbon materials using locally available satoyama resources. Specifically, we have demonstrated the production of biomass‐derived graphitic carbons from softwood‐derived chips and Japanese cedar via low‐temperature catalytic graphitization, followed by their application as LIB anodes [75, 76]. In addition, we have investigated the use of binchotan (white charcoal) derived from satoyama forests as an SIB anode material, confirming its potential as a negative electrode [77]. Notably, our synthesis approach enables the formation of highly crystalline carbon at relatively low temperatures, offering a pathway toward energy‐efficient production of battery‐grade carbon materials. Ongoing work focuses on further optimizing these processes to improve both structural control and electrochemical performance. Finally, while LIBs and SIBs offer high energy and power, their flammable organic electrolytes pose safety risks. This has led to proposals for alternative energy storage devices that use wood‐derived activated carbons (ACs) with aqueous (nonflammable) electrolytes, combining sustainable electrodes with inherently safer electrolytes.

These studies are technologically important, but they also show why satoyama biomass should not be judged only against battery‐grade graphite: the stricter the target microstructure, the more feedstock heterogeneity and process control become critical.

**FIGURE 5:**
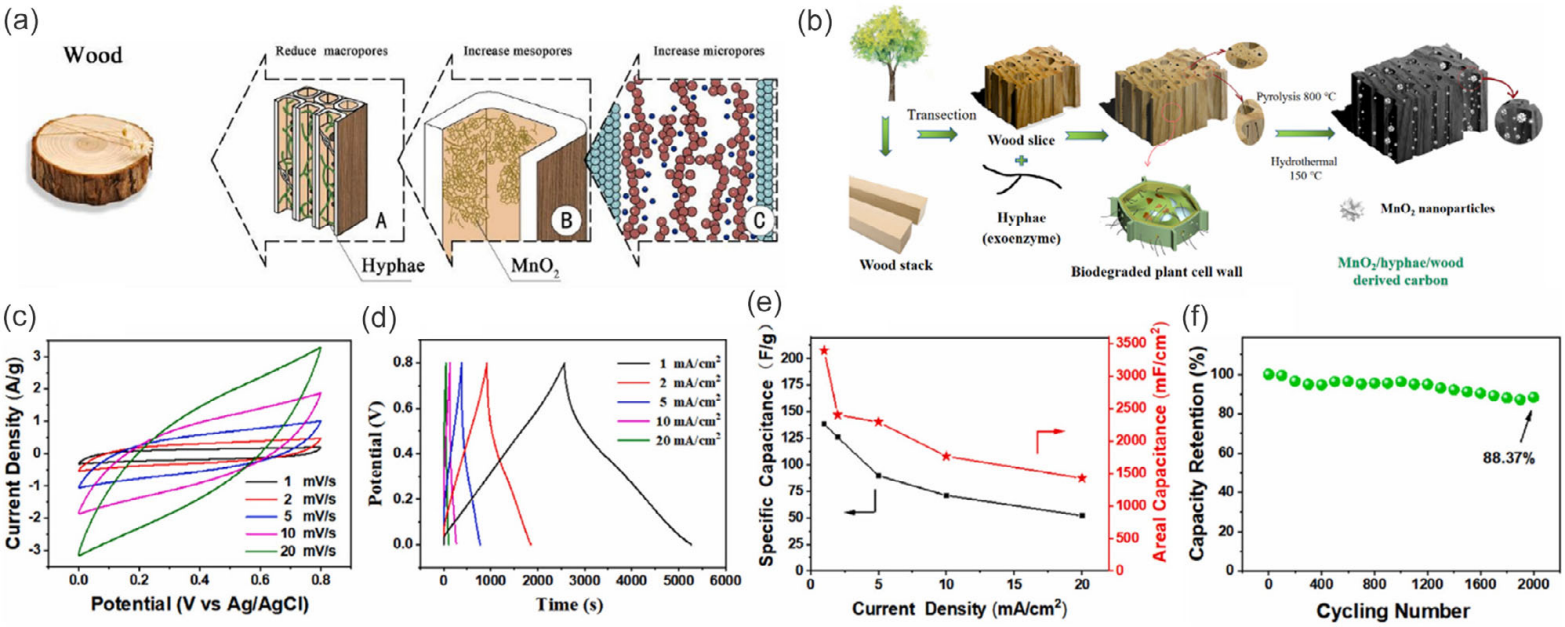
Schematic representations of: (a) the synergistic effect of fungal pretreatment and pseudocapacitive material (MnO_2_) on multiscale pore regulation in wood and (b) synthesis of the MnO_2_/hyphae/wood‐derived carbon (HWC‐3M) electrodes. Electrochemical performance of HWC‐3 M: (c) CV curves at different scan rates; (d) Galvanostatic charge–discharge curves at different current densities; (e) rate performance; and (f) cycling performance. Images were adapted from a previous study [32] with permission from Elsevier, Copyright 2023.

Supercapacitors using carbon materials with high surface area and superior electrical conductivity, such as ACs, carbon aerogels, carbon nanotubes, and graphene‐based materials, hold great promise as stationary energy storage devices [78]. In fact, carbon materials with high surface areas and electrical conductivity can be utilized to generate very high power and large capacitances [79, 80]. Supercapacitors are used in a wide range of electrolyte systems, including aqueous electrolytes such as H_2_SO_4_ and KOH, organic electrolytes such as acetonitrile and propylene carbonate, and ionic liquids. On one hand, a water‐based supercapacitor is a candidate for energy storage from an economical and environmental viewpoint. However, aqueous electrolytes are constrained by the stability window of water, so considerable effort has been devoted to expanding the practical cell voltage [81, 82, 83]. In this context, woody biomass‐derived ACs are suitable as raw materials from various perspectives, and numerous efforts are currently underway [31, 84, 85]. In supercapacitors, mesopores and micropores play a major role in ion storage [86]. For example, Zhang et al. [31] reported a wood‐derived thick carbon electrode that delivered 285.6 F g^−1^ and an energy density of 38.0 mWh cm^−3^ in an aqueous supercapacitor configuration. Separately, He et al. [87] systematically analyzed how component stripping, micropore development, and surface functional groups influence the electrochemical behavior of wood‐derived electrodes. As shown in Figure 5a, Cao et al. [32] used fungal pretreatment to reconstruct the multiscale pore network of paulownia‐derived carbon before MnO_2_ loading. After carbonization of the pretreated wood at 800°C, the aqueous supercapacitor electrode was fabricated by supporting MnO_2_ on the wood‐derived carbon under hydrothermal conditions, as shown in Figure 5b. The aqueous supercapacitor exhibited 3,395 mF cm^−2^ at a current density of 1.0 mA cm^−2^ and good cycling stability (retention of 88.6% after 2,000 cycles), as shown in Figure 5c–f [32].

Because conventional supercapacitors excel in power density but remain limited in energy density, hybrid configurations that incorporate organic redox species have attracted increasing interest [88]. Quinone compounds are particularly attractive redox‐active materials because they can be synthesized from natural resources, making energy‐storage devices based on quinone‐impregnated electrodes a rational and sustainable choice. Once the quinone loading exceeds a certain threshold, distinct charge–discharge plateaus emerge, indicating a transition from typical electric double‐layer capacitive behavior toward behavior more characteristic of rechargeable organic batteries than of conventional supercapacitors. In this context, lignin, a redox‐active organic component of woody biomass, has also been directly utilized in battery fabrication [89]. Moreover, when quinone‐based molecules are used as the active species, microbial conversion pathways may provide a route to producing such compounds from wood‐derived resources [90].

Motivated by these possibilities, our research group has focused on the development of aqueous all‐organic rechargeable batteries employing quinone monomers. In an early study, Katsuyama et al. [30] achieved an energy density of 13 Wh kg^−1^ and 91% capacity retention after 1,000 cycles by impregnating quinones into oak‐derived activated carbons. We subsequently extended this concept to more practical cell architectures by fabricating aqueous all‐organic cells using realistic current collectors and electrode thicknesses [91].

Later, we further addressed both manufacturing and materials‐design challenges, including the development of electrode‐sheet fabrication processes [92] and clarification of the correlation between the micropore size of biomass‐derived activated carbons and the molecular size of the impregnated organic species, which proved critical to electrochemical performance [93].

These studies collectively shifted our work from proof‐of‐concept quinone impregnation toward the engineering of a practical aqueous all‐organic battery platform, culminating in pouch cells with exceptionally high organic loading and minimal performance decay even after 3,000 charge–discharge cycles [93].

### 3.2 Electrocatalytic and Bioelectrochemical Energy Conversion

In this Account, ‘energy conversion’ refers to electrode‐mediated conversion processes such as the hydrogen evolution reaction (HER), oxygen reduction reaction (ORR), and bioelectrochemical reactions in microbial fuel cells.

**FIGURE 6:**
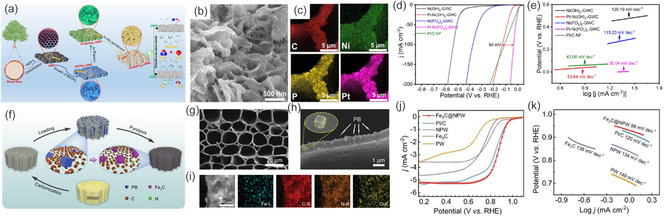
(a) Schematic illustration of the preparation procedure of the Pt‐Ni(PO_3_)_2_‐GWC bifunctional nanocatalyst for energy‐saving H_2_ production. (b) High‐magnification SEM image and (c) elemental mappings of Pt‐Ni(PO_3_)_2_‐GWC after long‐term stability test for the HER. (d) Polarization curves after iR correction of various catalysts in 1.0 M KOH. (e) Corresponding Tafel plots obtained from the linear sweep voltammetry (LSV) curves. Images in (a–e) were adapted from a previous study [33] with permission from Elsevier, Copyright 2023. (f) Construction of Fe_3_C@NPW. (g) Overview SEM image of PB@PW. (h) Magnified SEM image of PB@PW. (i) High‐angle annular dark‐field scanning transmission electron microscopy and corresponding energy dispersive X‐ray spectroscopy scanning transmission electron microscopy elemental mapping images of iron, carbon, nitrogen, and oxygen elements in Fe_3_C@NPW; (j) ORR LSV curves, and (k) Tafel curves obtained from LSV curves in (j) for Fe_3_C@NPW, Fe_3_C, Pt/C, NPW, and PW. Images in (f–k) were reproduced in part from a previous study [34] with permission from Wiley, Copyright 2022.

Although electrode catalysts for electrocatalytic water splitting are primarily composed of metals and alloys, wood‐derived carbons have recently been investigated as supporting materials for HER and OER catalysts [33, 94, 95, 96, 97]. By using wood‐derived carbon support, it is possible to obtain high HER and OER activity without using high‐cost carbon nanotubes or graphene. Figure 6a shows that after creating a highly crystalline carbon platform by impregnating wood with Ni(Ac)_2_ and then performing catalytic graphitization, a catalyst supporting nickel phosphate and platinum was prepared (Pt‐Ni(PO_3_)_2_‐GWC). Based on the scanning electron microscope (SEM) images and elemental mapping (Figure 6b,c), nickel and platinum remained uniformly dispersed throughout the graphitic carbon after the long‐term stability test for HER. Based on the linear sweep voltammetry measurements shown in Figure 6d,e, this catalyst exhibits excellent HER activity, requiring only 10 mV (overpotential) to achieve a current density of 10 mA cm^−2^ and a Tafel slope of 30 mV dec^−1^. It exhibits excellent durability for >120 h. High ORR activity in biomass‐derived carbons is typically achieved through heteroatom doping (e.g. N, S, or P) and/or Fe‐containing active sites that tune the local electronic structure and oxygen adsorption behavior [98, 99, 100]. Wood‐based electrodes for ORR are being developed [34, 101]. Figure 6f shows the procedure for fabricating an ORR catalyst in which wood carbonized at 900°C is impregnated with Prussian blue (PB) as a nitrogen and iron source under hydrothermal conditions and then carbonized again. PB nanocubes derived from a hybrid precursor and paulownia wood‐derived N‐doped carbon were denoted as PB@PW and Fe_3_C@NPW, respectively. Figure 6g,h show SEM images of carbonized wood impregnated with PB; thus, the existence of support for PB was confirmed. The high‐angle annular dark‐field scanning transmission electron microscopy and corresponding energy dispersive X‐ray spectroscopy scanning transmission electron microscopy analyses of Fe_3_C@NPW that were further supported and re‐annealed revealed that iron and nitrogen were dispersed throughout. A dual‐active‐site catalyst composed of Fe_3_C nanoparticles bonded to nitrogen‐doped carbon (Fe_3_C@NPW) derived from paulownia wood was fabricated using an active site binding strategy. One site on the Fe_3_C nanoparticles participates in activation of water molecules, and another site on the nitrogen‐doped carbon is involved in activating oxygen molecules. Benefiting from the synergistic effect of dual active sites, Fe_3_C@NPW exhibits remarkable catalytic activity for ORR, with a half‐wave potential of 0.87 V (vs. RHE) in alkaline electrolyte, which surpasses that of commercially available Pt/C. Furthermore, the zinc‒air battery (ZAB) assembled with Fe_3_C@NPW as the cathode catalyst achieves a high specific capacity of 804.4 mA h g_Zn_^−1^ and long‐term stability of 780 cycles. The solid‐state ZAB model also exhibits satisfactory performance with an open circuit voltage of 1.39 V and a high peak power density of 78 mW cm^−2^. These excellent performances are some of the best among nonnoble‐metal electrode materials. Minimally processed charcoal is also useful in microbial fuel cells and methanogen biocathodes, where stringent structural control is less critical and local carbon sources may be particularly practical [35, 36, 102, 103, 104].

Our group has also explored the development of ORR electrocatalysts using a doping strategy introduced during hydrothermal carbonization. In one of our early attempts, iron and nitrogen were incorporated into Douglas fir‐derived carbon within a hydrothermal reaction field to prepare an ORR electrocatalyst [105]. Although the resulting activity was not particularly high, this study provided an important starting point by showing that heteroatom and metal incorporation could be achieved during the hydrothermal conversion of woody biomass. More recently, we developed an ORR catalyst derived from rice husk, demonstrating that satoyama‐inspired local resources need not be limited to wood alone [106]. In this context, silica‐rich agricultural residues such as rice husk, rice straw, and bamboo may serve as useful complementary feedstocks to woody biomass, particularly when improved durability is required in composite carbon catalysts [107].

## 4 Green processes for reducing processing costs

In the current context, wood‐derived electrodes exhibit properties that are comparable to those of conventional materials. Typically, when producing carbon from wood, the commonly employed carbonization process is endothermic and demands elevated temperatures, resulting in considerable energy consumption. For converting biomass into carbon materials, hydrothermal carbonization (HTC) is attractive because it operates at lower temperatures than conventional pyrolysis‐type routes and can process wet feedstocks without a separate drying step [108]. Utilization of HTC reactions can facilitate exothermic reactions, atomic doping, and low‐temperature carbonization. As an example, by using a hydrothermal reaction field during catalytic graphitization for semi‐carbonization, our group succeeded in producing wood‐based graphite‐like carbons at low temperatures [75]. There are also examples of doping during semi‐carbonization [105]. A recent review on HTC‐derived carbons for sustainable electrochemical energy storage further emphasizes the relevance of wet‐processing routes for lowering process intensity and broadening biomass compatibility [108]. In addition, laser‐induced carbonization processes have been utilized in synthesis of highly conductive carbons [109]. Graphite production has also been achieved through laser carbonization. The process was extremely efficient at 0.41 g Wh**^‐^** ^1^ (2.44 Wh g**^‐^** ^1^), and the energy content of its byproduct (i.e., bio‐oil) exceeded that needed to power the process [28]. The process energy demand in graphitization was lower than that of the artificial graphite and natural graphite fabrication processes, as shown in Table 5. However, for large‐scale production, the need for precise, point‐by‐point laser processing remains a key scale‐up challenge.

**TABLE 5:** Process energy demand for artificial graphite (AG), natural graphite (NG), and biochar graphite (BCG).

Type of graphite	Process energy demand, Wh g^−1^
AG in graphitization [24]	6.17
NG in total processes [25]	10.8
AG in total processes [26]	12.8
BCG in graphitization [28]	2.44

When wood is used as a support material, the loading of metal particles into mesopores and micropores and the loading of organic materials become crucial considerations. Additionally, the incorporation of oxides and organic compounds into hybrid supercapacitors can benefit from supercritical CO_2_‐assisted impregnation into ACs. Employing supercritical CO_2_ in the impregnation process for ACs in organic batteries can result in cleaner and more efficient devices [110, 111]. Collectively, these technologies have the potential to greatly contribute to utilization of porous wood materials.

## 5 Summary and Outlook

In this Personal Account, I have argued that forest underuse in Japan can be reframed as an opportunity for nature‐positive materials engineering when satoyama stewardship is linked to carbon‐electrode design. I highlighted our work with biomass‐derived carbons in batteries, supercapacitors, and electrocatalytic systems – all aimed at shrinking mining footprints while enhancing biodiversity and strengthening regional value chains.

Looking ahead, several key challenges must be addressed: (i) reducing process energy requirements and costs; (ii) managing logistics and variability of biomass feedstocks; (iii) scaling up manufacturing while maintaining quality control; and (iv) developing robust metrics to verify ‘nature‐positive’ outcomes (improvements in biodiversity, landscape function, and avoided ecological harm). High performance alone is not sufficient. Region‐appropriate technologies should be prioritized only when they are evaluated rigorously for hidden environmental burdens, resource trade‐offs, and social consequences. In this sense, nature‐positive materials engineering requires both electrochemical performance and system‐level accountability. Table 6 contrasts conventional materials engineering with the nature‐positive materials engineering framework proposed here, which emphasizes locality, ecological compatibility, and distributed value creation. By shifting raw material inputs toward locally available, non‐mined resources and end‐use markets toward local or regional needs, I argue that a more nature‐positive paradigm of materials engineering can be realized.

**TABLE 6:** Comparison between Conventional Materials Engineering and Nature‐Positive Materials Engineering.

Aspect	Conventional materials engineering	Nature‐positive materials engineering

Resources	Externally sourced, often from outside the region/abroad	Local/community‐based
Production sites	Centralized factories	Regionally distributed production facilities
Markets	Primarily global	Local or regional
Technology holders	Large corporations and specialized engineers	Community members and cross‐sector collaborators
Core values	Efficiency, performance, and mass production	Sustainability and diversity

From this perspective, materials scientists can further recognize the potential and diversity of regional biomass resources and develop device technologies that accommodate those resources. Likewise, forestry professionals can continue to prioritize forest conservation while exploring novel uses of forest biomass that fit contemporary lifestyles and regional needs. By building energy devices from locally sourced, bio‐based materials, we can begin to envision a more decentralized, community‐driven model of materials production that remains connected to local ecosystems and resource cycles.

## Funding

This work was supported by Environmental Restoration and Conservation Agency (Grants JPMEERF20223C04, JPMEERF20223R02), and the collaborative research between Kyoto University and Tohoku University as part of a broader collaborative research framework involving Daicel Corporation.

## Conflicts of Interest

The author declares no conflicts of interest.

## Data Availability

The data that support the findings of this study are available from the corresponding author upon reasonable request.
